# Sex Differences in Nociceptin/Orphanin FQ Peptide Receptor-Mediated Pain and Anxiety Symptoms in a Preclinical Model of Post-traumatic Stress Disorder

**DOI:** 10.3389/fpsyt.2018.00731

**Published:** 2019-01-08

**Authors:** Yong Zhang, Ian Schalo, Cindy Durand, Kelly M. Standifer

**Affiliations:** ^1^Department of Pharmaceutical Sciences, College of Pharmacy, University of Oklahoma Health Sciences Center, Oklahoma City, OK, United States; ^2^Oklahoma Center for Neuroscience, University of Oklahoma Health Sciences Center, Oklahoma City, OK, United States

**Keywords:** allodynia, hyperalgesia, NOP receptor, PTSD, sex differences, single prolonged stress, anxiety, Nociceptin/Orphanin FQ

## Abstract

Nociceptin/Orphanin FQ (N/OFQ) is a neuropeptide that modulates pain transmission, learning/memory, stress, anxiety, and fear responses via activation of the N/OFQ peptide (NOP or ORL1) receptor. Post-traumatic stress disorder (PTSD) is an anxiety disorder that may arise after exposure to a traumatic or fearful event, and often is co-morbid with chronic pain. Using an established animal model of PTSD, single-prolonged stress (SPS), we were the first to report that NOP receptor antagonist treatment reversed traumatic stress-induced allodynia, thermal hyperalgesia, and anxiety-like behaviors in male Sprague-Dawley rats. NOP antagonist treatment also reversed SPS-induced serum and CSF N/OFQ increase and circulating corticosterone decrease. The objective of this study was to examine the role of the NOP receptor in male and female rats subjected to traumatic stress using Wistar wild type (WT) and NOP receptor knockout (KO) rats. The severity of co-morbid allodynia was assessed as change in paw withdrawal threshold (PWT) to von Frey and paw withdrawal latency (PWL) to radiant heat stimuli, respectively. PWT and PWL decreased in male and female WT rats within 7 days after SPS, and remained decreased through day 28. Baseline sensitivity did not differ between genotypes. However, while male NOP receptor KO rats were protected from SPS-induced allodynia and thermal hypersensitivity, female NOP receptor KO rats exhibited tactile allodynia and thermal hypersensitivity to the same extent as WT rats. Male NOP receptor KO rats had a lower anxiety index (AI) than WT, but SPS did not increase AI in WT males. In contrast, SPS significantly increased AI in WT and NOP receptor KO female rats. SPS increased circulating N/OFQ levels in male WT, but not in male NOP receptor KO, or WT or KO female rats. These results indicate that the absence of the NOP receptor protects males from traumatic-stress-induced allodynia and hyperalgesia, consistent with our previous findings utilizing a NOP receptor antagonist. However, female NOP receptor KO rats experience allodynia, hyperalgesia and anxiety-like symptoms to the same extent as WT females following SPS. This suggests that endogenous N/OFQ-NOP receptor signaling plays an important, but distinct, role in males and females following exposure to traumatic stress.

## Introduction

Sex differences in post-traumatic stress disorder (PTSD) and the presence of co-morbid pain are well documented in the clinic, but studies of sex differences in PTSD-related behaviors using animal models has been limited primarily to fear responses/processing and hippocampal plasticity [for review see (1)]. Literature from clinical studies suggests that females exhibit a higher reported rate of PTSD and longer lasting, more severe symptoms [for review see (1–4)]. They also differ from males in the PTSD criterion/parameters noted in their response to stress (5), and in the relationship between PTSD and appearance of co-morbid conditions such as depression, schizophrenia, cognitive decline, and pain (6). Therefore, it is important to study and understand these sex differences to facilitate development of more effective treatments.

A number of preclinical PTSD models exist; one of the most frequently utilized, and well-characterized preclinical models of PTSD that possesses content and criterion validity is single-prolonged stress [SPS; (7, 8)]. The SPS model reproduces physiological and psychological symptoms that appear unpredictably over time following a short period of severe trauma. This exposure is applicable to civilians confronted with natural disasters (e.g., tornadoes, fires, and earthquakes) and other terrifying situations, as well as military populations. SPS produces sustained neuroendocrine disruption (including enhanced negative feedback of the hypothalamic-pituitary-adrenal (HPA) axis), hyperarousal, fear extinction retention impairment, altered sleep-wake cycles, changes in the noradrenergic system, increased CRF in the brain, and reduced synaptic plasticity and BDNF that are all hallmarks of PTSD in humans (7–10). SPS also produces co-morbid depressive-like behaviors (11–13), enhanced alcohol reward (14), and cognitive impairment (15) in male rats. Ours was one of the first groups to report changes in nociceptive sensitivity in male rats as a co-morbid condition using a preclinical model of PTSD (16, 17). Since our initial report we, and other groups, confirmed and extended our findings of allodynia, hyperalgesia (18–22) and anxiety-like behaviors (11–13, 16) in males. We found only a single study that assessed changes in pain sensitivity in female rats following SPS. Female rats subjected to an enhanced SPS protocol (with foot shock) exhibited increased visceral sensitivity, but no comparisons to males were included (23).

One molecule that links the HPA axis, sensory systems and disease states is Nociceptin/Orphanin FQ (N/OFQ). N/OFQ is the endogenous ligand for the N/OFQ peptide (NOP) receptor, and also is known as ORL1 or KOR-3 (24–27). N/OFQ and the NOP receptor are widely expressed in the central nervous system (CNS), particularly in the forebrain and the descending pain pathway, which are involved in emotional and pain processing (28). N/OFQ bi-directionally modulates many key biological functions in the CNS that are impacted by PTSD and/or chronic pain (including nociceptive sensitivity, learning and memory, stress and anxiety and reward), via activation of the NOP receptor (29–32). This bidirectional modulatory pattern of N/OFQ often produces conflicting results that may vary between species, strains, time and method of drug administration, assay method and/or stress. For example, N/OFQ and other NOP agonists block stress-induced analgesia, but may produce analgesia in other instances (33–38). Serum and CSF of patients with acute and chronic pain contain elevated N/OFQ levels (39, 40). Similarly, serum and CSF from rats that exhibited allodynia and hyperalgesia following SPS contain higher N/OFQ levels than from sham-treated rats (17, 22). We subsequently reported that blockade of N/OFQ actions with a NOP antagonist prevented up-regulation of N/OFQ and anxiety-like symptoms and reversed SPS-induced allodynia and hyperalgesia, confirming a role for N/OFQ in modulation of pain sensitivity, anxiety and HPA axis modulation following traumatic stress (22).

A single nucleotide polymorphism (SNP) in the non-coding region of the NOP receptor gene was associated with PTSD symptoms in women that experienced severe traumatic stress (29), but we have no knowledge about how that SNP might alter NOP receptor expression, N/OFQ signaling or nociceptive sensitivity. While preclinical studies examining fear, hyperarousal, depression, and cognitive deficits in females exposed to traumatic stress have become more numerous (including sex differences in SPS-induced cued fear extinction retention deficits and hippocampal plasticity (41, 42), only a single study examining changes in nociceptive sensitivity in preclinical models of PTSD was found (23). Acquisition of NOP receptor gene knockout (KO) rats (ORL1-/-) (43, 44) enabled us to examine for the first time, the role of the N/OFQ-NOP receptor system in female rats following exposure to the SPS model of PTSD, and further examine its role in males.

## Methods

### Animal Treatment

Wild type (WT) Wistar Han rats were purchased from Charles River Labs (Wilmington, MA). Fourteen male homozygous Oprl1-TGEM® KO (referred to herein as ORL1^−/−^ or NOP “KO”) rats in Wistar Han background and a set of homozygous breeders were obtained from Transposagen (Lexington, KY). The rest of the KO animals were generated from the homozygous breeding pairs in the OUHSC animal facility; genotype was confirmed by Transnetyx (Cordova, TN). The animal protocol was approved by the University of Oklahoma Health Sciences Center's Institutional Animal Care and Use Committee. Studies conformed to the FASEB Statement of Principles for the use of animals in research and education. Research was compliant with the Animal Welfare Act Regulations and other Federal Statutes relating to animals and experiments involving animals, and adhered to the principles set forth in the Guide for Care and Use of Laboratory Animals. All experiments conformed to the guidelines of the International Association for the Study of Pain. Rats were acclimated to their environment for at least 7 days following arrival and housed in the animal facility under a 12-h light: 12-h dark cycle (lights on at 0600 h) with free access to food and water. Intact male (250–300 g) and female (175–210 g) KO and WT rats (9–10 weeks of age) were randomly divided into control or SPS groups (*N* = 33 total males and 37 total females; *N* = 7~10/group). **SPS** consists of complete restraint for 2 h, grouped forced swimming (*N* = 2–3 at a time) for 20 min, and exposure to diethyl ether until consciousness is lost. Once rats recovered from anesthesia, they were returned to their cages and left undisturbed for 7 days as previously described (8, 17).

**Nociceptive sensitivity** was assessed by measuring hind paw withdrawal threshold (PWT) from pressure and paw withdrawal latency (PWL) from radiant heat prior to SPS exposure and every 7 days thereafter through day 28. An electronic von Frey anesthesiometer (IITC Life Sciences, Inc., Woodland Hills, CA) was utilized for mechanical/tactile nociception assessment. Rats were placed in clear plastic boxes with a wire mesh floor, and acclimated for 15–20 min. PWT was obtained from the mid-plantar aspect of the left hind paw. Approximately 1.5 h after PWT assessment, the wire mesh floor was replaced with a glass floor and rats acclimated for approximately 30 min. Then, a plantar analgesia meter (IITC Life Sciences, Inc., Woodland Hills, CA) was utilized to measure PWL to an infrared light beam directed toward the left hind paw with the lamp set at 25% active intensity as previously described (17). Cutoff time was set at 30 s to prevent tissue damage. The average of three sets of scores (taken 5 min apart) was the PWT/PWL for each rat, each week. Decrease in PWT compared to control rats was termed allodynia since the intensity of the pressure applied was dynamic. Decrease in thermal sensitivity compared to control rats was termed hyperalgesia because all rats were exposed to same heat intensity and only latency was determined. All behavioral assessments were made between 0900 and 1200 h. An algesia index was determined for each treatment group by calculating the area under the PWL or PWT curve from y = 0 up to the PWL/PWT at each time point, using GraphPad Prism. Sex and stress group differences were determined by two-way analysis of variance (ANOVA).

The presence of anxiety-like symptoms was assessed using the **elevated plus maze (EPM) test** on day 9 post-SPS (and again on day 30 for female rats) as previously described (17). The plus maze consisted of two open (50 × 10 cm) and two closed (50 × 10 × 40 cm) arms elevated 40 cm above floor with average light levels 40–55 lux. Each rat was placed in the center of the apparatus with its head facing a closed arm, and activity was recorded for 5 min from the center of the rat body. The apparatus was cleaned with 30% ethanol between each recording session. Recording sessions were analyzed with Any-maze software (Stoelting Co., Wood Dale, IL) for mobile/immobile time, traveled distance, arm entries, and time spent in arms. The anxiety index (AI) was calculated as described (45): 1—[(% time in open arms + % entries into open arms)/2]. Female rats were exposed to the EPM a second time on day 30 post-SPS (3 week interval between testing). Rats do not develop habituation or sensitization to the EPM when testing intervals are spaced at least 3 weeks apart (46, 47).

### Collection of Fluid and Tissue Samples

Rats were euthanized by injection with Beuthanasia (i.p. 0.22 mg/kg, Schering-Plow Animal Health, Union, NJ, USA) between 1,200 and 1,600 h on day 28–30 post-SPS. Blood was withdrawn from the heart with an 18-gauge needle, and kept at room temperature for 30 min before centrifugation at 5,000 × g, 4°C for 5 min when serum was collected. CSF (150 ~ 200 μl) from each rat was withdrawn by inserting a 26-gauge needle into the cisterna magna. Lumbar spinal cord was removed and the dorsal horn dissected and collected. All samples were stored at −80°C until biochemical analysis was performed.

### Radioimmunoassay (RIA)

N/OFQ levels in 50 μL of serum or CSF were determined in duplicate by RIA (Phoenix Pharmaceuticals, Belmont, CA) according to the protocol suggested by the manufacturer. Total amount of N/OFQ immunoreactivity (IR) was calculated and expressed as pg/mL. Samples that fell outside of the range of the standard curve or that were contaminated with blood were not included; specific information is provided in the figure legend. The individual conducting each assay was blind to the grouping.

### ^35^S-GTPγS Binding

Spinal cord (SC) dorsal horn membranes from male and female WT and KO rats were prepared and assayed for N/OFQ stimulation of ^35^S-GTPγS binding as previously described to examine NOP receptor activity (22). Briefly, tissue was homogenized in 1 mL ice-cold TED buffer (5 mM Tris-HCl, 1 mM EDTA, 1 mM DTT, pH 7.4) containing 10% (w/v) sucrose and centrifuged at 1,000 × g for 10 min. The supernatant was washed twice by centrifugation at 9,000 × g for 20 min and resuspended in 1 mL of TED buffer. The suspension was kept on ice for 30 min, followed by centrifugation at 35,000 x g for 10 min. The pellet was stored at −80°C until use. Membrane protein, 10 μg as determined by BCA assay, was incubated at 25°C for 60 min in plastic tubes containing a total volume of 100 μL: 0.5% bovine serum albumin, 0.1% bacitracin, 10 μM GDP, 0.3 nM ^35^S-GTPγS, 1 mM EDTA, 1 mM DTT, 5 mM MgCl_2_, 100 mM NaCl and 10^−9^−10^−5^ M N/OFQ. The reaction was terminated by rapid filtration through glass fiber filters using a Brandel cell harvester. Radioactivity was determined by liquid scintillation spectroscopy. Non-specific binding was measured in the presence of 100 μM unlabeled GTPγS, which was subtracted from total binding to define specific ^35^S-GTPγS binding.

### Data Analysis

Data Analysis and graph preparation were performed using GraphPad Prism 7.01 software (GraphPad Software, La Jolla, CA, USA). Data are expressed as mean ± SD unless indicated otherwise. Statistical comparisons of behavioral and neurochemical data were performed by two-way ANOVA with *post-hoc* analyses as automatically recommended by the software. Tukey's multiple comparisons tests were used when comparing every row (or column) mean with every other row (or column) mean. Sidak's was used to determine differences within columns or rows. Results were considered statistically significant if *P* < 0.05. All data were subjected to D'Agostino & Pearson (*N* > 8) or Shapiro-Wilk (*N* < 8) normality tests prior to analysis. Those groups that failed the normality test (*p* < 0.05) were subjected to an outlier test (ROUT; Q = 1%), as recommended (48) to determine if the outlier was responsible for the failed normality test. If exclusion of outlier(s) led to passing the normality test and altered statistical result, the exclusion was made. If it did not alter the statistical outcome, no data were excluded from that group. The 6 samples that were excluded by outlier test are listed in the appropriate figure legend. Pearson's Correlation Analysis was performed with the following data aligned from each rat: D7 and D28 PWT and PWL, D9 (and D30 in females) anxiety index and % time in open arms, serum N/OFQ and CSF N/OFQ. Correlations were made with data from Control and SPS-treated rats of each sex and genotype.

## Results

### Nociceptive Sensitivity

**Figure 1:**
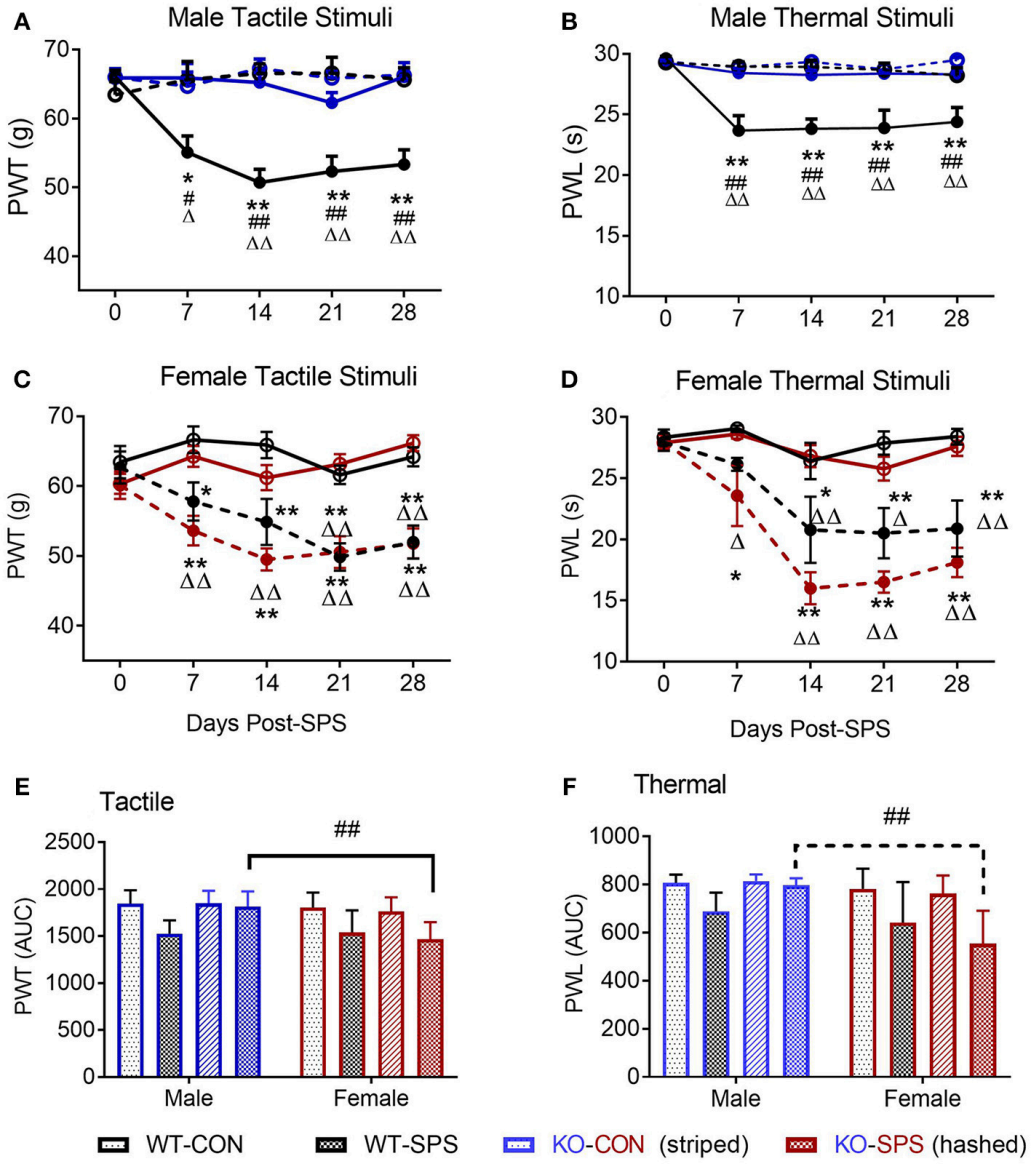
NOP receptor KO genotype prevented development of tactile allodynia and thermal hyperalgesia in male, but not female, rats following SPS. Rats were randomly assigned into groups within their genotype and sex. For males **(A,B)** Wild type (WT)-CON (*n* = 7; black open circles), WT-SPS (*n* = 7; solid black circles) and ORL1-/- (KO): CON (*n* = 9; blue open circles), and KO-SPS (*n* = 10; solid blue circles). For Females **(C,D)** WT-CON (*n* = 9), WT-SPS (*n* = 9) and KO-CON (*n* = 10; red open circles), KO-SPS (*n* = 9; solid red circles). Mechanical **(A,C)** and Thermal **(B,D)** sensitivity was assessed prior to (day 0) and every 7 days following SPS. PWT and PWL data were analyzed by two-way ANOVA (Treatment/Time x Genotype) followed by Tukey's *post-hoc* analysis and data are presented as mean ± SEM. SPS induced allodynia and hyperalgesia in WT males as previously described, but KO male responses did not differ from WT or KO control rats (*p < 0.05 and **p < 0.01 for WT-CON vs. WT-SPS; ^Δ^*p* < 0.05 and ^ΔΔ^*p* < 0.01 for KO-CON vs. KO-SPS; #*p* < 0.05 and ##*p* < 0.01 for WT-SPS vs. KO-SPS). In contrast, female WT and KO rats developed allodynia and hyperalgesia in response to SPS (*p < 0.05 and **p < 0.01 for WT-CON vs. WT-SPS; ^ΔΔ^*p* < 0.01 for KO-CON vs. KO-SPS). No differences between female WT and KO SPS or between WT and KO Control groups appeared at any time point. The area under the time-nociceptive sensitivity curves (AUC) of WT and KO males and females generated in response to tactile **(E)** and thermal **(F)** stimuli of each treatment group are presented as mean ± SD, and were analyzed by two-way ANOVA for sex × SPS treatment. Significant *post-hoc* differences between sexes within each treatment group were determined with Sidak's multiple comparison test (##*p* < 0.01).

Our primary goal was to determine if SPS produces tactile allodynia and thermal hyperalgesia in male and female Wistar WT and NOP receptor KO rats. Male Wistar WT rats responded to SPS (closed black circles) by developing tactile allodynia (Figure 1A) and thermal hyperalgesia (Figure 1B) at the same rate and to the same extent as previously reported in Sprague-Dawley rats. This is reflected by decreased PWT and PWL, respectively, compared to WT Control (CON) rats (black open circles). Remarkably, PWTs and PWLs of NOP KO rats subjected to SPS (solid blue circles) did not differ from those of their untreated control littermates (blue open circles), indicating that the absence of the NOP receptor protected the rats from SPS-induced allodynia and hyperalgesia. Two-way ANOVA indicated that there was a significant interaction between time and genotype-stress treatment for tactile (A: [*F*_(12, 145)_ = 3.143, *p* = 0.0002]) and thermal (B: [*F*_(12, 145)_ = 4.176, *p* < 0.0001]) stimuli. PWT and PWL in WT and KO unstressed rats did not differ from each other at any time point. Tukey's multiple comparisons test revealed WT male SPS rats differed from WT control (^*^p < 0.05 and ^**^p < 0.01), KO Control (^Δ^*p* < 0.05 and ^ΔΔ^ *p* < 0.01) and KO SPS (##*p* < 0.05 and ## *p* < 0.01).

Similar to WT males, WT female rats also developed tactile allodynia (Figure 1C) and thermal hyperalgesia (Figure 1D) following SPS (solid black circles). However, unlike the males, absence of the NOP receptor did not protect female KO rats from developing allodynia or hyperalgesia following SPS (solid red circles). Female NOP KO rats developed allodynia and hyperalgesia to the same extent as WT females, but no differences in nociceptive sensitivity were noted between female WT CON (open black circles) and KO CON (open red circles) rats. Two-way ANOVA indicated significant interaction between Time x Genotype-stress treatment for tactile (C: [*F*_(12, 165)_ = 2.925, *p* = 0.0011]) and thermal stimuli (D: [*F*_(12, 165)_ = 3.139, *p* = 0.0005]). Tukey's multiple comparisons test indicated SPS-treated WT and KO rats differed from WT and KO controls. Pearson correlation analysis revealed that there was a significant correlation between D28 PWT and PWL in both WT and KO female rats (Table 1). The correlation just missed significance in WT males (*p* = 0.05).

**Table 1:** Pearson correlation analysis in male and female WT and NOP receptor KO rats.

**Pairwise correlations**	**Male WT**	**Male KO**	**Female WT**	**Female KO**
D28 PWT:PWL		
Pearson r *P*-value	0.531 0.05	−0.082 0.739	0.76 <0.001^*^	0.90 <0.001^*^
Serum N/OFQ:D28 PWT		
Pearson r *P*-value	−0.609 0.027^*^	0.186 0.475	0.207 0.410	−0.432 0.073
Serum N/OFQ:D28 PWL		
Pearson r *P*-value	−0.102 0.740	−0.198 0.447	0.409 0.092	−0.288 0.246
CSF N/OFQ:D28 PWL		
Pearson r *P*-value	0.281 0.331	−0.244 0.381	−0.801 0.001^*^	−0.475 0.073
CSF N/OFQ:D9 Anxiety Index		
Pearson r *P*-value	−0.427 0.128	−0.305 0.269	0.736 0.004^*^	−0.384 0.157
CSF N/OFQ:D7 PWT		
Pearson r *P*-value	0.037 0.900	−0.396 0.144	−0.651 0.016^*^	0.003 0.993
CSF N/OFQ:D7 PWL		
Pearson r *P*-value	0.045 0.879	−0.138 0.623	−0.591 0.033^*^	0.280 0.311
D9 Anxiety Index: D28 PWT		
Pearson r *P*-value	−0.171 0.560	0.241 0.32	−0.484 0.042^*^	0.479 0.071
D9 Anxiety Index: D28 PWL		
Pearson r *P*-value	−0.519 0.057	0.149 0.543	−0.418 0.084	−0.322 0.117
D9 Anxiety Index: D9 % Open Arm time		
Pearson r *P*-value	−0.92 <0.001^*^	−0.945 <0.001^*^	−0.86 <0.001^*^	−0.904 <0.001^*^
CSF N/OFQ: D9 % Open arms time		
Pearson r *P*-value	0.583 0.029^*^	0.303 0.273	−0.580 0.038^*^	−0.282 0.242
D30 Anxiety Index: D28 PWT		
Pearson r *P*-value	ND	ND	−0.576 0.012^*^	−0.216 0.374
D30 Anxiety Index: D28 PWL		
Pearson r *P*-value	ND	ND	−0.841 <0.001^*^	−0.176 0.470
D30 Anxiety Index: D9 Anxiety Index		
Pearson r *P*-value	ND	ND	0.318 0.199	0.467 0.044^*^

*ND, not determined*.

* *Indicates a significant correlation*.

To directly examine this apparent sex difference, an algesia index for each group for each stimuli was generated by calculating its area under each treatment group's time-nociceptive sensitivity curve (AUC; Figures 1A–D). A two-way ANOVA of the calculated AUC was performed with sex as the row effect and traumatic stress for each genotype (treatment) as the column effect (Figures 1E–F).There was a significant interaction between sex and treatment for tactile (E: [*F*_(3, 62)_ = 4.005; *P* = 0.0114]) and thermal (F: [*F*_(3, 62)_ = 5.063; *P* = 0.0034]) stimuli. Sidak's multiple comparison's test compared the effect of sex within each group. The only *post-hoc* sex difference noted was between males and females in the KO SPS groups. Females in the KO SPS group were more sensitive to tactile (*p* < 0.001) and thermal (*p* < 0.001) stimuli than males in that treatment group.

### Anxiety-Like Behaviors

**Figure 2:**
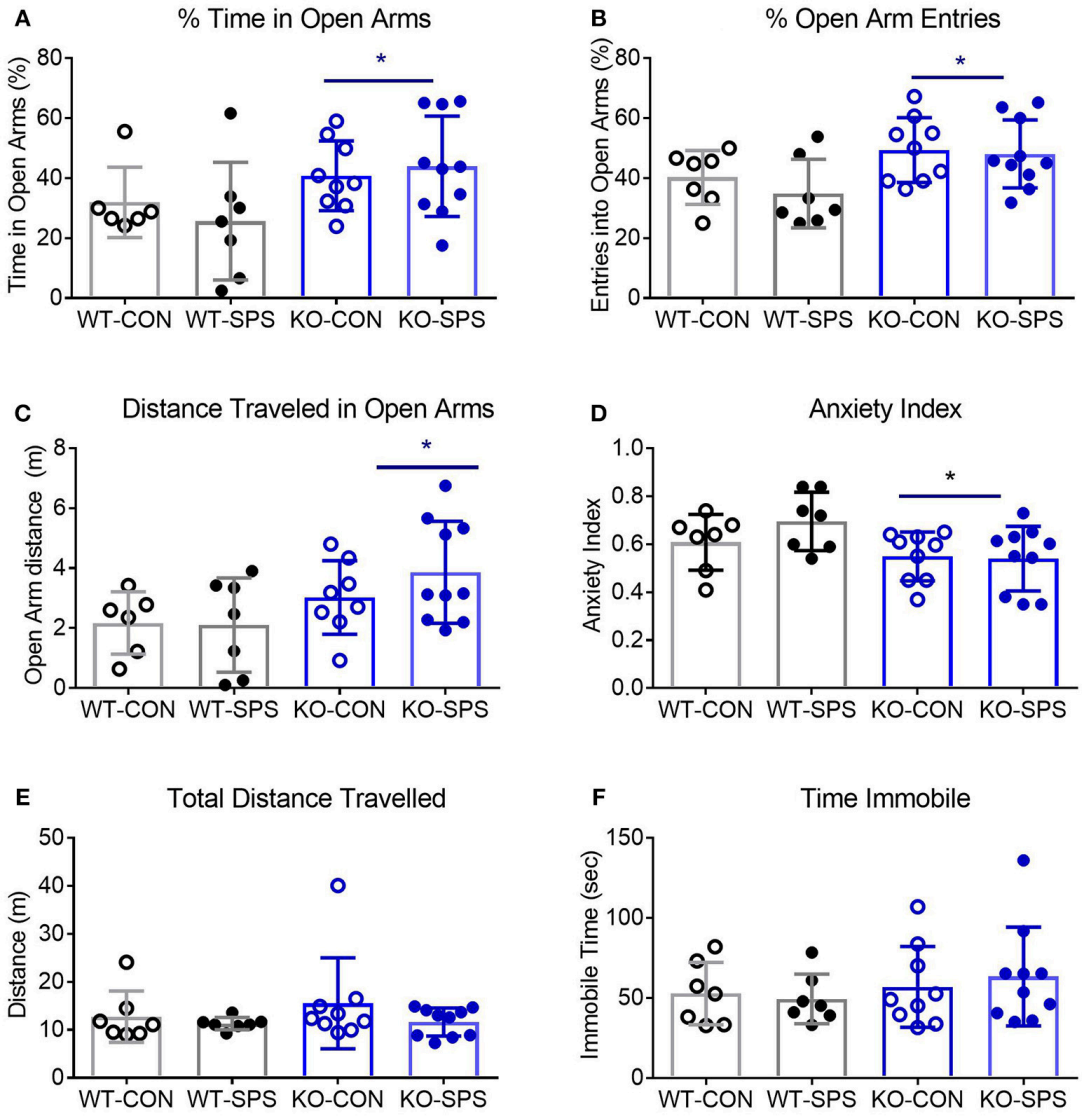
Male NOP receptor KO rats exhibited significantly fewer anxiety-like symptoms than WT rats. Data of individual parameters **(A–F)** were analyzed by two-way ANOVA and presented as mean ± SD. WT male rats did not exhibit a significant increase in anxiety-like behaviors following exposure to SPS **(A–D)**, though the Anxiety Index **(D)** trended in that direction. However, there was a significant effect of genotype (*p < 0.05) on % time in open arms **(A)**, % open arm entries **(B)**, Distance traveled in open arms **(C)** and anxiety index **(D)**. Rats in KO-CON and KO-SPS groups spent a greater % of time in open arms, made a greater % of entries into open arms, covered a greater distance in the open arms **(C)**, and had lower anxiety indexes **(D)** than WT rats in either treatment group. Tukey's *post-hoc* analysis revealed no *post-hoc* differences. There was no significant effect of genotype or treatment on total distance traveled **(E)** or immobile time **(F)**, confirming that rat mobility, per se, was not responsible for any differences noted. Outliers (Q = 1%): one rat each was excluded from the WT-CON group in panel A and WT-CON and KO-CON in panel C by ROUT. KO-SPS group in panel F failed the normality test, but no outliers were identified.

**Figure 3:**
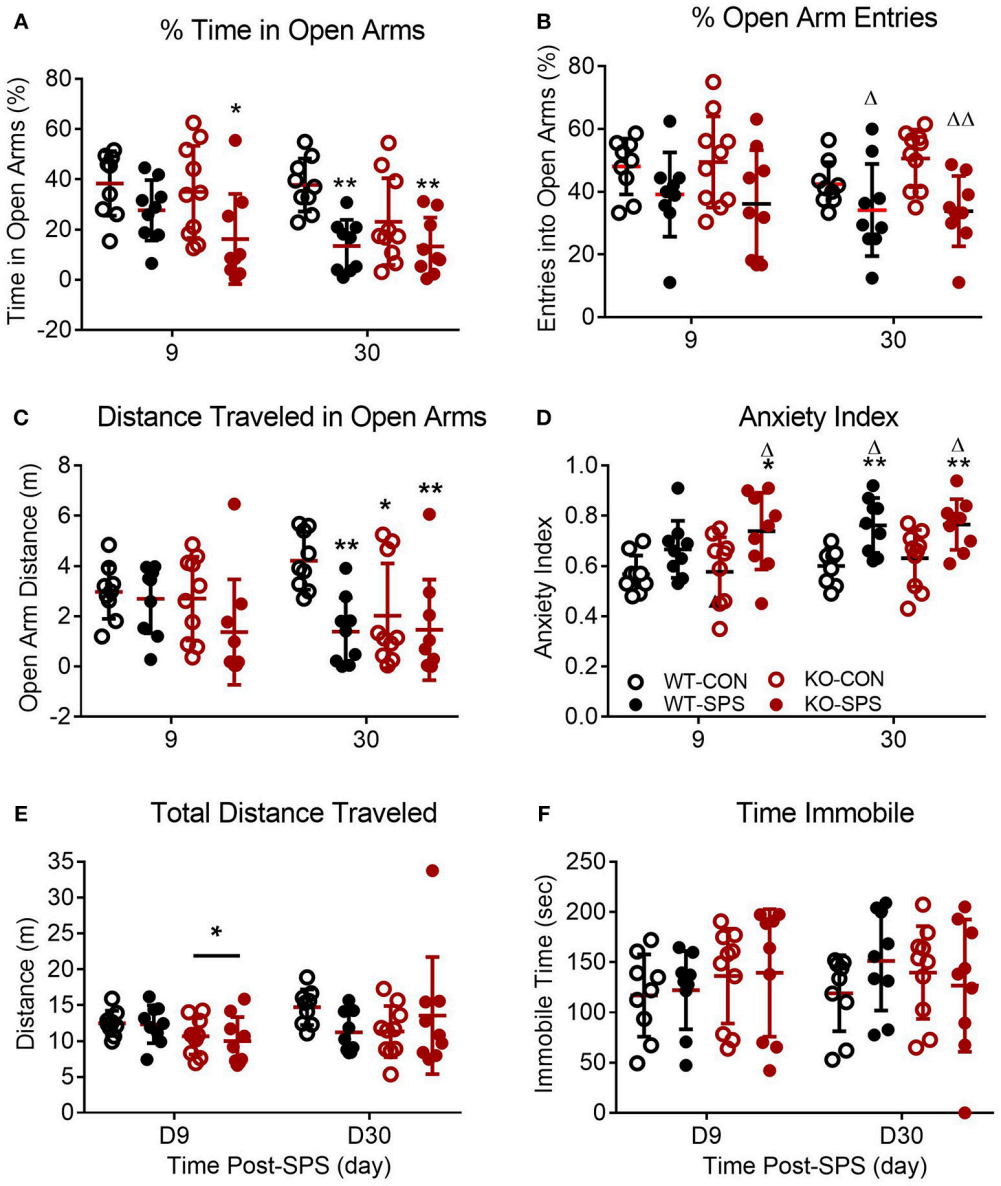
Female WT and KO rats develop anxiety-like symptoms following SPS. Data of individual parameters **(A–F)** measured at days 9 and 30 post-SPS were analyzed by two-way ANOVA with Tukey's *post-hoc* analysis (genotype x SPS treatment) and presented as mean ± SD. Unlike male rats, there was a significant effect of SPS treatment on four parameters tested (see Table 2 for F values). *Post-hoc* analysis confirmed that female rats exhibited a significant increase in anxiety-like behaviors at one or both time points following exposure to SPS (designated by *p < 0.05 and **p < 0.01 vs. WT-CON; ^Δ^*p* < 0.05, and ^ΔΔ^*p* < 0.01 vs. KO-CON) in % time in open arms **(A)**, % open arm entries **(B)**, distance traveled in open arms **(C)** and anxiety index **(D)**. Rats in SPS-treated groups spent a smaller percentage of total time in open arms, made fewer % of all arm entries into open arms, traveled a shorter distance while in open arms **(C)** and exhibited higher anxiety indexes **(D)**. However, no significant effect of SPS was noted for total distance traveled **(E)** or time immobile **(F)**. Unlike male rats, no significant effect of genotype was noted for any parameter except a transient effect on total distance traveled at day 9 (**E;** *p < 0.05).

The secondary goal of this study was to determine if SPS produces anxiety-like symptoms in male and female Wistar WT and NOP receptor KO rats. The appearance of anxiety-like symptoms at day 9 post-SPS was assessed using the elevated plus maze (EPM) and analyzed by two-way ANOVA for genotype x traumatic stress for males (Figures 2A–F; Table 2) and females (Figures 3A–F; Table 2). Unlike the previous report in SD rats, no significant interaction or significant effect of SPS was found for any anxiety-like behavior in male Wistar rats (Figures 2A–F; Table 2). However, a significant effect of genotype was noted for % time in open Arms (**A**), % open arm entries (**B**), distance traveled in open arms (**C**), and anxiety index (AI; **D**). There was no effect of genotype on total distance traveled (**E**) or immobile time (**F**). Both groups of male KO rats appeared less “anxious” in that they spent more time, made more entries into and traveled a longer distance within the open arms, and scored a lower anxiety index than WT CON and WT SPS rats, but no differences between treatment groups were detected by *post-hoc* analysis.

**Table 2:** Elevated plus maze ANOVA values and sources of variation from comparisons of genotype and SPS treatment on anxiety-like behaviors in male and female WT and NOP receptor KO rats.

		**Males**		**Females**			
		**Day 9**		**Day 9**		**Day 30**	
**Parameter**	**Source of variation**	**F (DFn, DFd)**	***P*-value**	***F* (DFn, DFd)**	***P*-value**	***F* (DFn, DFd)**	***P*-value**
% Time in open arms	SPS		NS	(1, 33) = 8.17	0.007*	(1, 33) = 16.1	< 0.001*
	Genotype	(1, 27) = 6.0	0.021*		NS		NS
% Open arm entries	SPS		NS	(1,33) = 5.96	0.02*	(1, 33) = 12.25	0.001*
	Genotype	(1, 27) = 8.67	0.006*		NS		NS
Distance traveled in open arms	SPS		NS		NS	(1, 33 = 9.05	0.005*
	Genotype	(1, 29) = 6.07	0.02*		NS		NS
Anxiety index	SPS		NS	(1, 33) = 10.3	0.003*	(1, 33) = 20.4	P < 0.001*
	Genotype	(1, 29) = 6.46	0.017*		NS		NS
Total distance traveled	Genotype		NS	(1, 33) = 5.73	0.023*		NS
Time immobile	None						

*NS, not significant*.

Unlike their male counterparts (Figure 2), genotype played no role in anxiety-like behaviors in female rats (Figures 3A–F, Table 2). However, there was a significant effect of SPS in female rats at day 9 (Table 2), with *post-hoc* analysis indicating that female KO-SPS rats spent significantly less % of time in open arms (**A**; *p* < 0.05) and had a higher AI (**D**, *p* < 0.05) than untreated rats. Since female rats exhibited significant anxiety-like behaviors at day 9, we extended the observation of anxiety-like symptoms by EPM for 3 weeks later (Day 30). Significant effects of SPS were noted on all four parameters at day 30 (Figures 3A–D; Table 2). *Post-hoc* differences between groups in response to SPS became more pronounced over time as SPS-treated WT and KO rats spent a smaller fraction of time (**A**; *p* < 0.01) and traveled a shorter distance while in open arms (**C**; *p* < 0.01) compared to WT CON; open arm entries were a smaller percentage of all arm entries compared to KO CON at day 30 (**C**; *p* < 0.05 for WT SPS and *p* < 0.01 for KO-SPS**)**. The AI increased in both SPS-treated groups at day 30 compared to WT (*p* < 0.01) and KO-CON (*p* < 0.05) rats (Figure 3D). There was a transient effect of genotype on total distance traveled at day 9 but not day 30 (Figure 3E; Table 2), but no individual differences were noted between groups by *post-hoc* analysis. Similar to nociceptive sensitivity in females, no effect of genotype or SPS was noted on either day of immobile time in female rats (Figure 3F). A direct analysis of sex differences between anxiety index at day 9 between males and females by two way ANOVA with Sidak's multiple comparisons indicates a significant interaction between sex (**[***F*_(3, 62)_ = 3.687, *P* = 0.0165]) and treatment group ([*F*_(3, 62)_ = 3.19, *P* = 0.0297]). Within each treatment group the only sex difference was found in the KO SPS group (^**^p < 0.01).

Pearson's correlation analysis of anxiety-like behaviors, PWT and PWL revealed a significant negative correlation between D9 AI and PWT in female WT rats that was absent in female KO rats (Table 1). A similar trend for D9 AI and PWL that just missed significance was noted for male (*p* = 0.057) and female WT (*p* = 0.084) rats; no trend was noted in KO rats. The negative correlation indicates that the anxiety index increases as PWT or PWL decrease (more sensitive to nociceptive stimuli). The same correlation between pain and anxiety was found in WT, but not KO, females with D30 AI and PWT (*p* = 0.012) and PWL (*p* < 0.001).

### N/OFQ RIA

**Figure 4:**
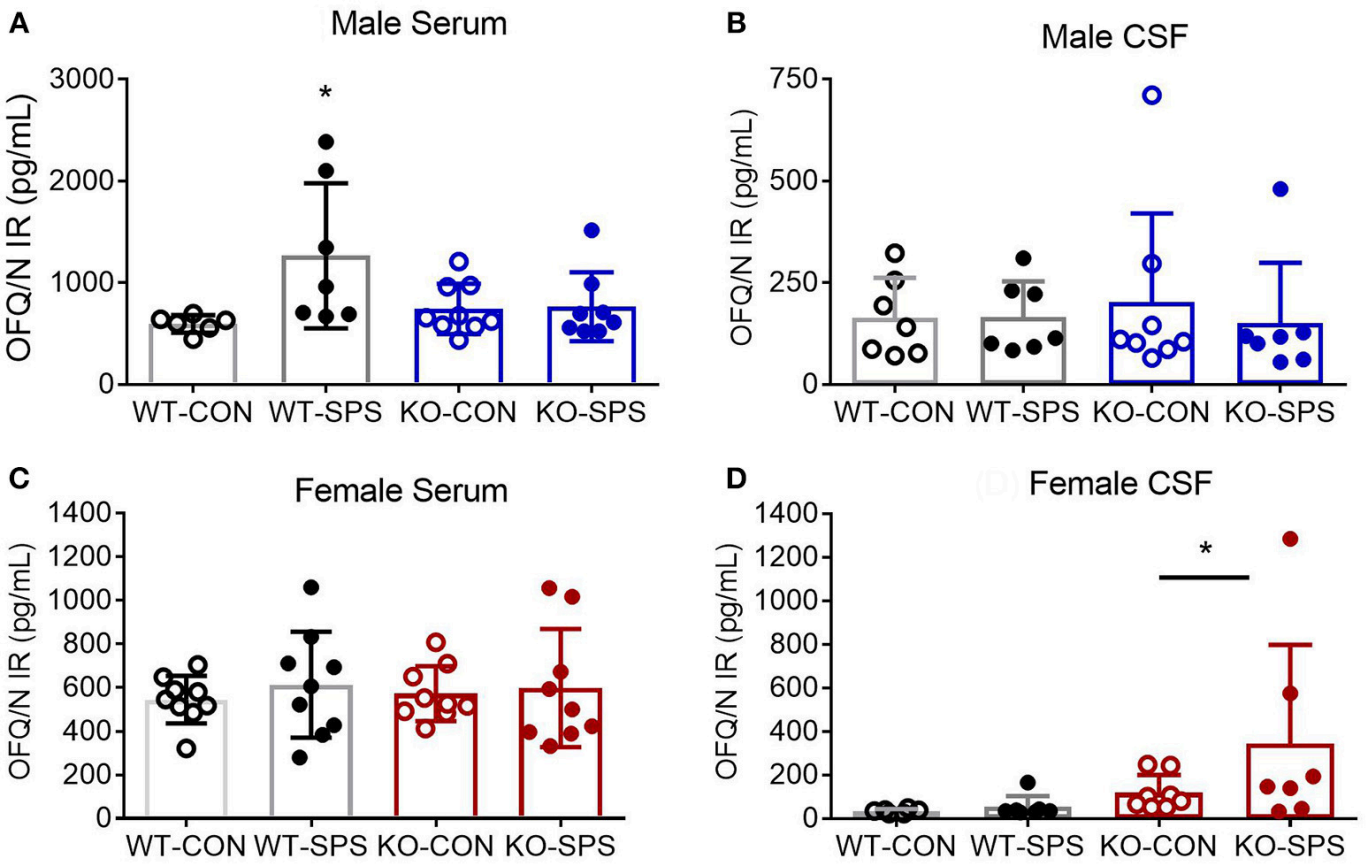
Knockout of NOP receptor prevented SPS-induced increase in serum N/OFQ levels in male rats, but SPS did not change serum N/OFQ levels in female rats or CSF N/OFQ levels in males or females. Serum **(A,C)** and CSF **(B,D)** samples collected at day 28 (males: **A,B**) or 30 (females: **C,D**) after SPS were assessed for N/OFQ content using RIA; analysis was by two-way ANOVA with Tukey's *post-hoc* analysis and error bars represent mean ± SD. SPS increased serum N/OFQ in male WT (*p < 0.05), but not KO, rats. There was no significant effect of SPS on CSF N/OFQ levels in male WT or KO rats **(B)**. Sample sizes were smaller than expected because several samples were excluded. The value of one serum sample from a male WT-CON was found to be an outlier by ROUT (Q = 1%) and values from two male KO-SPS rats fell outside the range of the standard curve and could not be included. For CSF **(B)**, two values were excluded for being out of range (1 each in KO-CON and KO-SPS), and two were excluded from KO-SPS for being contaminated with blood. Both male CSF KO groups failed normality tests. No significant differences in serum N/OFQ between the four groups of female rats was found **(C)**. However, a significant effect of genotype for CSF N/OFQ levels was revealed (**D**, *p < 0.05). Exclusions for female rats in serum samples included one each in WT-CON and WT-SPS for being out of range and one from KO-CON for blood. CSF exclusions for female rats include: WT-CON one out of range and one by ROUT (Q = 1%); WT-SPS one out of range and one by ROUT (Q = 1%), and two female rats each in KO-CON and KO-SPS groups for being out of range of the standard curve (2) or the presence of blood in the samples (2). Both female CSF KO groups failed normality tests.

Serum and CSF samples were collected at the end of the experiment (28–30 days post-SPS) to quantify levels of N/OFQ by RIA. SPS significantly increased serum N/OFQ in male WT Wistar rats (^*^p < 0.05), but had no effect on N/OFQ levels in NOP KO rats (Figure 4A). There was a significant interaction between genotype and SPS ([*F*_(1, 26)_ = 4.553; *P* = 0.0425]) and a significant effect of SPS ([*F*_(1, 26)_ = 5.209, *p* = 0.0309]) by two-way ANOVA with Tukey's *post-hoc* test. Somewhat surprisingly, no significant effect of SPS or genotype was noted for CSF N/OFQ (Figure 4B) in males. RIA results of serum and CSF samples from female rats euthanized at day 30 post-SPS found no significant effects of genotype or stress on serum N/OFQ (Figure 4C). Analysis of CSF N/OFQ from female rats revealed a significant effect of genotype [*F*_(1, 24)_ = 4.564, *p* = 0.0431], but no differences between groups were noted with *post-hoc* analysis (Figure 4D). To directly compare sex differences in levels of serum and CSF N/OFQ in the presence and absence traumatic stress, data from males and females were analyzed by two-way ANOVA for sex × treatment group, with Sidak's multiple comparisons test. We found a significant interaction between sex and treatment group [*F*_(3, 58)_ = 2.85, *p* = 0.0451], and significant effects of sex [*F*_(1, 58)_ = 11.53, *p* = 0.0014] and treatment group [F _(3, 58)_ = 3.999, *p* = 0.0117]. *Post-hoc* analysis indicated that male WT SPS was significantly different from female WT SPS (*p* < 0.01). The same analysis was performed with CSF but no significant interaction or effects of sex or treatment group was noted.

Sex differences also were noted in a number of pairwise correlations involving N/OFQ (Table 1). Serum N/OFQ negatively correlated with PWT in WT males, but not in KO males or WT females; KO females trended toward a negative correlation between serum N/OFQ and PWT (*p* = 0.073). However, it was CSF N/OFQ levels that negatively correlated with PWT and PWL in WT females, with no correlation noted in males or KO females. WT males (^*^p = 0.029) and WT females (^*^p < 0.037) both showed significant correlations between CSF N/OFQ levels and % time in open arms (Table 2). However, the correlation in females was negative (*r* = −0.580), as noted with PWT and PWL while the correlation in males was positive (0.583). Thus, in females increased CSF N/OFQ levels were associated with less % of time in open arms (more anxiety), while increased CSF N/OFQ levels in males corresponded to greater % time in open arms (less anxiety). No significant correlations of CSF N/OFQ and % time in open arms were noted in KO rats.

### NOP Receptor-Mediated ^35^S-GTPγS Binding

**Figure 5:**
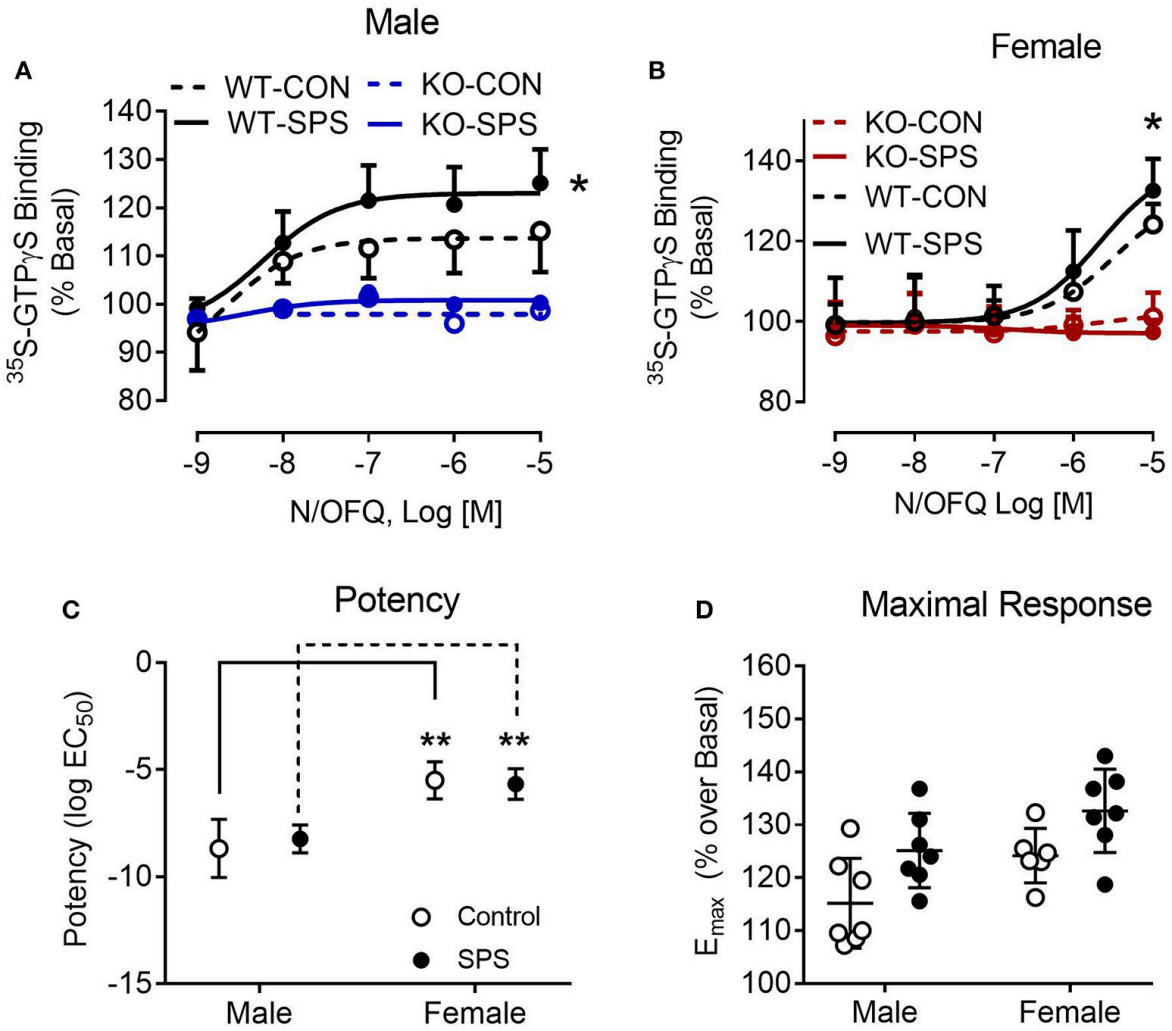
N/OFQ efficacy increases with SPS in dorsal spinal cord (SC) membranes from Wistar Han WT male **(A)** and female **(B)** rats; no efficacy found in SC membranes from KO rats. ^35^S-GTPγS binding was performed in the presence and absence of increasing concentrations of N/OFQ in spinal cord membranes from WT and KO male and female rats from sham control and SPS-treated groups; data are presented as mean ± SD. N/OFQ efficacy significantly increased following SPS in membranes from WT male (**A**: *p = 0.0285; *n* = 7 per group) and female (**B**: *p = 0.0354; *n* = 6–7) rats, but no measurable increase in basal ^35^S-GTPγS binding was noted with N/OFQ in membranes from male or female KO rats as determined by two-way ANOVA (genotype x stress) with a *post-hoc* Sidak's multiple comparison test (effect of SPS within each genotype). These results are consistent with the genotyping results and extend that analysis to confirm that no functional NOP receptors are expressed in the KO rats. Direct analysis of sex differences in potency **(C)** and efficacy **(D)** were performed using two-way ANOVA with Sidak's multiple comparison test. There was a significant effect of sex on potency [*F*_(1, 23)_ = 62.12, *p* < 0.001] in both CON and SPS tissues (*p* < 0.001). Though significant effects of sex [*F*_(1, 23)_ = 8.5–3, *p* = 0.0078] and SPS [*F*_(1, 23)_ = 10.6, *p* = 0.0035] were noted for efficacy, no sex-specific post-hoc differences were found between CON or SPS groups **(D)**.

To provide functional validation of NOP receptor loss and further examine N/OFQ efficacy following traumatic stress, N/OFQ-stimulated ^35^S-GTPγS binding in spinal cord dorsal horn membranes from male and female WT and KO rats was assessed (Figure 5). No response to N/OFQ was noted in membranes from KO male or female rats, further confirming the functional loss of NOP receptors in those animals. The efficacy (E_max_) of N/OFQ to elicit ^35^S-GTPγS binding in the spinal cord of WT male rats subjected to SPS significantly increased (*p* = 0.0285) compared to efficacy in WT CON (Figure 5A). Two-way ANOVA revealed significant effects of genotype [*F*_(1, 24)_ = 60.16, *p* < 0.001] and traumatic stress [*F*_(1, 24)_ = 4.673, *p* = 0.0408]. A similar increase in E_max_ also was noted in female WT rats following SPS (*p* = 0.03). Two-way ANOVA revealed a significant interaction between genotype and traumatic stress [*F*_(1, 22)_ = 6.831, *p* = 0.0159] and a significant effect of genotype [*F*_(1, 22)_ = 156.1, *p* < 0.001]. However, N/OFQ potency for eliciting the response was 500–1000-fold less in WT females (3.1 and 2.1 μM for Control and SPS, respectively) than in WT male rats (2.1 nM Control and 5.9 nM SPS). Direct analysis of sex differences in potency (Figure 5C) and efficacy (Figure 5D) were performed using two-way ANOVA of WT data only (since there was no effect in KO). There was a significant effect of sex on potency ([*F*_(1, 23)_ = 62.12, *p* < 0.001] in both CON and SPS tissues (*p* < 0.001 by Sidak's multiple comparison test). The same type of two-way ANOVA was performed to determine sex differences in efficacy. Though significant effects of sex [*F*_(1, 23)_ = 8.5–3, *p* = 0.0078] and SPS [*F*_(1, 23)_ = 10.6, *p* = 0.0035] were noted, no sex-specific E_max_ *post-hoc* differences were found within either the CON or SPS groups (Figure 5D).

## Discussion

Stress-induced analgesia has been well-documented, however considerable evidence suggests that acute and chronic stress produce hyperalgesia, including in the SPS model (17, 19–23, 49). The goal of this study was to evaluate the role of the NOP receptor in the development of allodynia, hyperalgesia, and anxiety symptoms in male and female rats following exposure to traumatic stress using WT and NOP receptor KO rats. As anticipated, results from male NOP receptor KO rats confirm the contribution of the NOP receptor to the development of tactile allodynia and thermal hyperalgesia following SPS reported earlier with a NOP receptor antagonist (22). The most important and novel finding of the study was that unlike the KO genotype effect in males, both wild type and NOP receptor KO female rats developed allodynia and hyperalgesia to the same extent as the wild type males. Loss of the NOP receptor afforded no protection to females from SPS-induced nociceptive hypersensitivity or anxiety-like symptoms. This is the first report of traumatic stress-induced effects on tactile and thermal nociceptive sensitivity changes over time in female rats, and the first to examine the role of N/OFQ-NOP receptor system in that process.

Significant sex differences also were noted in anxiety-like behaviors following SPS in WT and NOP receptor KO rats. Unlike our previous EPM results in male Sprague-Dawley (SD) rats, we did not find a significant effect of traumatic stress on anxiety-like behaviors in WT Wistar Han males. The cause of this difference is unclear. It may result from differences in strain, age and/or housing conditions. In our previous studies, after SPS the SD rats were housed alone, with dividers between cages to prevent visual contact. Changes in our cage racks precludes the use of dividers now, though unpublished studies with Sprague-Dawley rats housed in the new cages continue to exhibit elevated anxiety-like behaviors. The rats in this study were 2 weeks older when SPS was initiated compared to previous studies, due to extended quarantine upon initial receipt of the KO rats. Therefore, that age was used for all rats in this study. Instead of finding SPS-induced effects on anxiety-like behaviors in the male rats, we consistently found an effect of genotype on anxiety-like behaviors. Male KO rats in CON and SPS-treated groups showed fewer anxiety-like behaviors, including anxiety index. This did not result from sedative effects or impaired mobility as there was no difference in total traveled distance by stress or by genotype. Our results were not consistent with previous findings in untreated NOP receptor KO rats (44), where loss of the NOP receptor was anxiogenic compared to WT. Differences between control groups in the two studies may reflect the inherent variability of EPM from one lab to the next or from differences in the number of animals housed per cage (50). Rizzi et al. housed 3-4 NOP receptor KO rats per cage, while ours were housed 1-2 per cage. N/OFQ KO mice (another model that lacks a functional N/OFQ-NOP receptor system) housed with multiple mice/cage were anxiogenic compared to WT mice, but individually housed N/OFQ KO mice were anxiolytic compared to WT (50).

Unlike the male rats that exhibited no significant effect of traumatic stress on any anxiety-like behavior, both WT and NOP receptor KO female rats developed anxiety-like behaviors by day 9 following SPS (significant effects of traumatic stress). While the clinical data indicate that PTSD symptoms in human females tend to be more pronounced and last longer (4), the limited studies using PTSD models with adult female rats (including SPS) suggests that females are more resilient to PTSD symptoms (41, 51). To determine if female anxiety-like symptoms change over time with SPS, female rats also were assessed for the appearance of anxiety-like symptoms on day 30 post-SPS (Figure 3). Previous work suggested that a 3 week interval in combination with a different environment prevented the decrease in open arm exploration that often is noted in a second EPM test (46). In our study the WT females exhibited identical activities in open arms and anxiety index between day 9 and 30, indicating that open arm exploration was not affected in the repeated test after 3 weeks. The significant effect of traumatic stress was underscored by day 30 post-SPS when more group differences because apparent by *post-hoc* analyses than noted at day 9. Besides females rats developing significant SPS-induced anxiety-like symptoms and males not exhibiting increased anxiety, a direct comparison of sex differences in anxiety index at day 9 indicated that significant sex differences were found only within the SPS KO treatment group, with female AI greater than males.

The role of N/OFQ and the NOP receptor in modulation of anxiety-like symptoms in males and females remains an open question. Numerous studies cite anxiolytic and anxiogenic actions of N/OFQ and other NOP agonists (for review see (32) in male rats. However, administration of two different NOP antagonists decreased anxiety-like symptoms in male SD rats exposed to two different models of traumatic stress (22, 52), without altering spontaneous locomotion. Similar gender differences in anxiety and fear-related behaviors also were reported in N/OFQ KO mice (50).

Assessment of serum and CSF N/OFQ levels in male and female rats 28 and 30 days post-SPS, respectively, revealed several interesting findings. First, serum N/OFQ also was significantly elevated in WT male rats following SPS as noted previously, but not in the SPS KO males. However, no significant increase in CSF N/OFQ following SPS in WT or KO male Wistar rats was detected, which differs from previous studies in WT SD rats (17, 22). These results are consistent with increased levels of circulating N/OFQ following traumatic stress that acts on the NOP receptor to increase nociceptive sensitivity; the absence of the NOP receptor protects male rats from allodynia and hyperalgesia.

Interestingly, serum N/OFQ levels were not significantly increased by SPS in female WT or KO rats; serum N/OFQ levels in WT SPS male rats were significantly higher than levels in female WT SPS rats (Figure 4E). Similar to males, no significant differences in CSF N/OFQ levels were noted between treatment groups in females, but there was a significant effect of genotype (Figure 4D). Expression of the N/OFQ-NOP receptor system is modulated by female sex hormones, glucocorticoids, inflammatory mediators, and activity of the N/OFQ-NOP receptor system (53–57). This modulation is supported by the interaction between genotype and SPS on serum N/OFQ levels in males and significant effects of genotype on CSF N/OFQ levels in females. Interpretation of the CSF N/OFQ RIA results in this study was limited by greater than usual reductions in sample sizes per group due to difficulty obtaining clear CSF samples from the Wistar rats, and numerous samples being outside of the range of the standard curve of the RIA. Unfortunately, limited amounts of CSF obtained per rat precludes having enough sample left to re-assay if dilution or increased sample volume is required. There may have been subtle differences between groups that were masked by the large variability and smaller sample sizes.

We previously reported that N/OFQ efficacy at the NOP receptor in the dorsal lumbar spinal cord was increased in SPS-treated male Sprague-Dawley rats compared to controls (58), and similar results were noted with WT male and female Wistar rats in this study. However, we found significant sex differences in potency of N/OFQ for activation of GTPγS; N/OFQ was 1000-fold more potent in males than in females in this study. This suggests that NOP receptors in the dorsal spinal cord of female rats were desensitized. Spinal N/OFQ antinociception is gender specific: female rat sensitivity to spinal N/OFQ varies depending on estrogen state (59–62). For instance, while i.t. N/OFQ alleviated mustard oil-induced hyperalgesia in male, ovariectomized (OVX) and diestrous female rats, it did not reverse hyperalgesia in OVX + estradiol or proestrous female rats (60). We did not screen the females for stage of the estrous cycle in this study. However, a previous study of fear extinction and retention in female SD rats following SPS may provide some insight. In that study, even though all females were in diestrus when they were exposed to SPS (42), by day 9 post-SPS there were equivalent numbers of rats in each stage of the estrus cycle in each treatment group. It is clear that the overall effect of traumatic stress in the current study was to produce hyperalgesia and allodynia in WT and KO females regardless of estrus cycle stage, and in KO rats independent of the NOP receptor.

N/OFQ modulates nociceptive sensitivity through supraspinal (33, 63), spinal (59–64), and peripheral nerve sites (65, 66). Supraspinal N/OFQ inhibits stress-induced analgesia and produces hyperalgesia (33, 35, 63), consistent with SPS-induced allodynia and hyperalgesia. N/OFQ activates the HPA axis following acute administration or mild stress (67–69): HPA activation by N/OFQ resembled acute stress and was blocked by NOP antagonist treatment. Though acute stress reduced N/OFQ content in the brain, N/OFQ levels were restored within 24 h (67), suggesting that stress causes release and synthesis of endogenous N/OFQ. Acute or repeated social defeat stress also elevates NOP receptor mRNA in the brain, supporting the hypothesis that dysfunction of the N/OFQ system contributes to behavioral and hormonal dysregulation following stress (69).

In mice, i.c.v. N/OFQ blocked stress-induced antinociception (SIA) equally in males and females (33). Though levels of N/OFQ did not significantly increase in CSF from WT male or female rats subjected to SPS in this study, it is possible that the lack of NOP receptor in the male KO rats ensured that stress-induced analgesia remained intact, and that could explain the protective effect of the NOP receptor KO in males. This was clearly not the case with female rats following SPS. CSF N/OFQ levels negatively correlate with PWT, PWL and % open arms time, and positively correlate with anxiety index in WT females. This is consistent with increased sensitivity to tactile and thermal stimuli, less time in open arms and a higher anxiety index in those WT females with the highest levels of CSF N/OFQ. The correlation was absent in KO females, as one might expect since no NOP receptors were available to interact with N/OFQ. However, the KO females experienced the same extent of allodynia, hyperalgesia and anxiety like behaviors as the WT females, indicating that activation of NOP receptors is not the only mechanism by which those behaviors are mediated.

Though spinal N/OFQ has generally been found to produce analgesia, very low levels of N/OFQ (femtomole) administered i.t. produced thermal hyperalgesia and tactile allodynia in male mice (70, 71). Thus, low levels of N/OFQ in the CSF following SPS also may contribute to hyperalgesia in males, and this hyperalgesia would be lost in the NOP receptor KO rats. The potency of N/OFQ for spinal NOP receptors in WT females was likely too weak to activate NOP receptors to modulate spinal nociceptive sensitivity, which is consistent with the lack of protection afforded NOP receptor KO females.

The third site at which N/OFQ has been found to alter nociceptive sensitivity is in the periphery at primary afferent nerve endings, where it increases nociceptive sensitivity via PLC/IP3-mediated release of Substance P (66). Levels of N/OFQ in the serum arise from white blood cells (65, 72, 73). Elevated serum N/OFQ in WT-SPS rats may contribute to hyperalgesia and allodynia through actions on peripheral nerve endings. While loss of the NOP receptor is sufficient to block hyperalgesia and allodynia in male rats, it is not sufficient to alleviate hyperalgesia and allodynia in female rats. There is strong evidence that sex differences in the immune system and in hormonal modulation of immune cells account for differences in chronic pain or pain sensitivity between males and females (74). This is likely the case for traumatic stress-induced allodynia, and future studies will address that possibility.

Our results confirm that circulating N/OFQ-mediated NOP receptor signaling in male rats plays an important role in modulating nociceptive sensitivity and serum N/OFQ levels following traumatic stress. Absence of NOP receptor expression prevented the development of tactile allodynia, thermal hyperalgesia and increased serum N/OFQ in male, but not female, rats following traumatic stress. However, loss of the NOP receptor in females did not alter behavioral or biochemical changes in response to SPS compared to WT controls. SPS-induced significant anxiety-like behavior in female, but not male, NOP receptor KO rats that persisted for at least 30 days. It occurred concomitantly with hyperalgesia and allodynia, and was correlated with CSF N/OFQ levels. Such dramatic differences in males and females in response to NOP receptor loss requires additional study to better understand the role of the N/OFQ-NOP receptor system in stress-induced pain modulation and the development of co-morbid PTSD symptoms such as allodynia, hyperalgesia and anxiety-like behaviors.

## Author Contributions

The study was written and conceived by KS and YZ. YZ, IS, and CD conducted the research. YZ and KS analyzed the data. The manuscript received input and was edited by all authors.

### Conflict of Interest Statement

The authors declare that the research was conducted in the absence of any commercial or financial relationships that could be construed as a potential conflict of interest.
